# Cation Exchange Membranes Coated with Polyethyleneimine and Crown Ether to Improve Monovalent Cation Electrodialytic Selectivity

**DOI:** 10.3390/membranes11050351

**Published:** 2021-05-10

**Authors:** Shanshan Yang, Shuaijun Yu, Lu Yu, Yuanwei Liu, Junbin Liao, Jiangnan Shen, Congjie Gao

**Affiliations:** 1 College of Chemical Engineering, Zhejiang University of Technology, Hangzhou 310014, China; yangss97@163.com (S.Y.); finesnow2021@163.com (S.Y.); yulu8823@163.com (L.Y.); jbliao@zjut.edu.cn (J.L.); 2 Department of Chemical Engineering and Safety, Binzhou University, Binzhou 256600, China; hbgfz@163.com; 3 Center for Membrane and Water Science & Technology, Zhejiang University of Technology, Hangzhou 310014, China; gaocj@zjut.edu.cn

**Keywords:** electrodialysis, monovalent cation permselectivity, codeposition, polyethyleneimine, 4′-aminobenzo-12-crown-4

## Abstract

Developing monovalent cation permselective membranes (MCPMs) with high-efficient permselectivity is the core concern in specific industrial applications. In this work, we have fabricated a series of novel cation exchange membranes (CEMs) based on sulfonated polysulfone (SPSF) surface modification by polyethyleneimine (PEI) and 4′-aminobenzo-12-crown-4 (12C4) codeposited with dopamine (DA) successively, which was followed by the cross-linking of glutaraldehyde (GA). The as-prepared membranes before and after modification were systematically characterized with regard to their structures as well as their physicochemical and electrochemical properties. Particularly, the codeposition sequence of modified ingredients was investigated on galvanostatic permselectivity to cations. The modified membrane (M-12C4-0.50-PEI) exhibits significantly prominent selectivity to Li^+^ ions ($P_{{\mathrm{Mg}}^{2+}}^{{\mathrm{Li}}^{+}}$ = 5.23) and K^+^ ions ($P_{{\mathrm{Mg}}^{2+}}^{K^{+}}$ = 13.56) in Li^+^/Mg^2+^ and K^+^/Mg^2+^ systems in electrodialysis (ED), which is far superior to the pristine membrane (M-0, $P_{{\mathrm{Mg}}^{2+}}^{{\mathrm{Li}}^{+}}$ = 0.46, $P_{{\mathrm{Mg}}^{2+}}^{K^{+}}$ = 1.23) at a constant current density of 5.0 mA·cm^−2^. It possibly arises from the synergistic effects of electrostatic repulsion (positively charged PEI), pore-size sieving (distribution of modified ingredients), and specific interaction effect (12C4 ~Li^+^). This facile strategy may provide new insights into developing selective CEMs in the separation of specific cations by ED.

## 1. Introduction

With the development of the economy and degradation of the environment, water crisis has become a worldwide problem. Considerable attention has been focused on the ion exchange membrane (IEM) in water purification, brackish-water desalination, demineralization, and wastewater treatment to meet green processes [1,2,3,4,5]. Furthermore, in specific water treatment processes, the development of IEMs with high-efficient permselectivity between counter-ions of different valences or the same valences is of significant importance, such as lithium extraction from high Mg^2+^/Li^+^ ratio salt-lake brine [6], acid recovery in hydrometallurgy, and the removal of F^−^ or NO_3_^−^ from groundwater [7,8].

In regard to cation exchange membrane (CEM), improving the permselectivity of monovalent cations can be achieved generally through three physicochemical effects: pore-size sieving, electrostatic repulsion, and specific interaction effects [5,9]. Following the pore-size sieving effect, the improvement of cross-linking degree [10,11] and formation of hydrophilic/hydrophobic separated structures [12,13,14] are the main approaches of altering the structures of membrane matrix to realize cation permselectivity. According to numerous reported studies, surface modification is considered as a facile and efficient approach to prepare monovalent cation permselective membranes (MCPMs). A series of cationic polyelectrolytes are usually utilized as functional modified materials, such as polypyrrole [15,16], polyaniline [17,18], polyethyleneimine (PEI) [19,20], polyquaternium-7 [21], chitosan, and its derivatives [22,23], which are decorated on the membrane surface through physical or chemical interaction.

Wang et al. reported a three-step modification scheme to covalently immobilize PEI multilayers on commercial heterogeneous CEMs, and the membranes presented superior monovalent selectivity in Na^+^/Mg^2+^ and H^+^/Zn^2+^ systems based on electrostatic repulsion effect [20]. Similarly, sulfonated poly(ether sulfone) (SPES) CEMs codeposited by PEI and dopamine (DA) presented 2.5 times higher permselectivity to H^+^ ions than SPES in a H^+^/Zn^2+^ system proposed by Li et al. [24]. Gohil et al. developed MCPMs by the oxidation polymerization of pyrrole on the CEM surface, and the tight and rigid polypyrrole layer achieved efficient separation of mono/bivalent cations [16]. In addition, Thakur et al. designed a novel modified method through controlled metal (Cu) loading on poly(2-acrylamido-2-methyl-propane-sulfonic acid) CEM by electro-less plating, and the optimized membrane exhibited extremely low Zn^2+^ leakage owing to the excellent pore-size sieving effect [25]. A series of CEMs modified by chitosan hydrochloride according to electrodepositing and coating respectively achieved high-efficient Na^+^/Mg^2+^ separation in optimized conditions, which depended on the synergetic effect of pore-size sieving and electrostatic repulsion [26]. Based on this synergetic effect, Pang et al. prepared novel modified sulfonated polyphenylsulphone (SPPSU) CEMs by in situ polymerization-deposited polyaniline followed by quaternizing with methyl iodide and the adjustment of positive charge density of the modification layer by controlling the degree of quaternization leading to prominent permselectivity for the Na^+^/Mg^2+^ and Li^+^/Mg^2+^ mixture [27]. In our previous work, the modification of a sulfonated polysulfone (SPSF) membrane through the codeposition of DA and 4′-aminobenzo-15-crown-5 (ACE) followed by GA cross-linking was beneficial to achieve pronounced K^+^ ions electrodialytic selectivity. Apart from pore-size sieving effect, the electric field driving and host–guest molecular recognition of ACE and K^+^ ions are essentially taken into account in evaluation of membrane permselectivity [28].

As a large ring organic compound with a polyether structure, crown ether possesses a nanoscale cavity structure, and it can bind specific metal ions to form a stable complex through electrostatic interactions [29,30], which can offer channels for specific ions. As is known, the cavity of 12-crown-4-ether (internal diameter 1.2–1.5 Å) just matches the diameter of a Li^+^ ion (1.2 Å) and they can form a 1:1 host–guest complexation [31,32]. Ali et al. reported a nanofluidic device for specific Li^+^ recognition via host–guest complexation between aminoethyl-benzo-12-crown-4 and Li^+^ in a confined environment [33]. In addition, PEI possesses a hyperbranched structure and abundant positively charged amine groups so that it is widely applied in membrane fabrication and modification [24,34].

Keeping in view the above findings, we proposed a novel modification means of combining positively charged PEI with 4′-aminobenzo-12-crown-4 (12C4) with the ability to recognize specific cations. Based on the inherent adhesion virtues of DA, sulfonated polysulfone (SPSF) CEMs were modified by the two-time codeposition of 12C4/DA and PEI/DA successively through Michael addition or Schiff base reaction [35], followed by the cross-linking of GA. The modified membranes have been characterized regarding the physicochemical and electrochemical properties. Furthermore, we have systematically investigated and analyzed the codeposition sequence of modified ingredients on galvanostatic permselectivity to cations in binary systems of Li^+^/Mg^2+^, K^+^/Mg^2+^, and K^+^/Li^+^.

## 2. Experimental Section

### 2.1. Materials

Sulfonated polysulfone (SPSF, the molar percent of bis(4–fluorophenyl) sulfone with respect to the total molar of difluoro monomers bis(4–fluorophenyl) sulfone and sulfonated bis(4–fluorophenyl) sulfone used in the synthesis procedure was 60%, Mw = 65,000, PDI = 2.25, Yanjin Technology Co., Ltd., Tianjin China), dopamine hydrochloride (98%, Aladdin Reagent Co., Ltd., Shanghai, China), benzo-12-crown-4 (98%, Meryer (Shanghai) chemical Technology Co., Ltd., Shanghai, China), polyethyleneimine (PEI, Mw = 10,000 Da, Aladdin Reagent Co., Ltd., Shanghai, China), glutaraldehyde (GA, 50% in water, Energy Chemical Co., Ltd., Shanghai, China), Tris(hydroxymethyl) aminomethane (Tris, 99.5%, Energy Chemical Co., Ltd., Shanghai, China), *N*,*N*-dimethylformamide (DMF, 99.5%), chloroform (99%), hydrogen peroxide (H_2_O_2_, 30% by weight), concentrated nitric acid (HNO_3_, 68%), acetic acid (HAc, 98%), lithium chloride (LiCl, 99.9%), potassium chloride (KCl, 99.5%), magnesium chloride (MgCl_2_, 99%), sodium sulfate (Na_2_SO_4_, 99.0%), and copper sulfate (CuSO_4_·5H_2_O, 99%) were received from Aladdin Reagent Co., Ltd. (Shanghai, China) and used without further purification. Deionized (DI) water was used throughout the experiments.

### 2.2. Membrane Fabrication

The procedures of SPSF-based CEM were carried out as follows: 10 g of SPSF was dissolved in 30 g of DMF solvent and mechanically stirred at 60 °C for 4 h. The homogeneous casting solution was cast on a glass plate with a thickness of 700 μm, which was followed by solvent evaporation at 60 °C. The obtained membrane was stored in DI water for use.

### 2.3. Surface Modification of the Membranes

4′-Aminobenzo-12-crown-4 (12C4) was synthesized according to the preparation of 4′-aminobenzo-15-crown-5 in our previously reported work [28]. PEI and 12C4 were respectively codeposited with DA by means of rapid deposition using CuSO_4_/H_2_O_2_ as a trigger [36]. In detail, PEI/DA codeposition solution was carried out as follows: 0.1 g of PEI, 0.2 g of dopamine hydrochlofide and 0.125 g of CuSO_4_·5H_2_O were dissolved in 100 mL of Tris–HCl buffer solution (pH ~8.5, 10 mM) followed by addition of 0.2 mL of H_2_O_2_. 12C4/DA codeposition solution was formed similar to PEI/DA solution. Instead of PEI, 12C4 was added in the aforementioned buffer solution with a designed molar ratio of 12C4/DA (0.50:1 and 0.75:1). 

**Figure 1:**
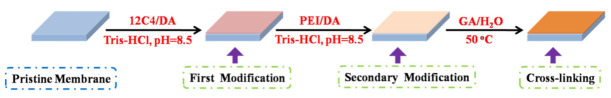
Schematic illustrations of the modified processes on SPSF membrane surface.

The modified processes on the membrane surface are shown in Figure 1. The circular pieces of CEMs with diameter of 6 cm were horizontally fixed in the holder. The upper surface of membrane was immersed into the fresh prepared 12C4/DA codeposition solution for 6 h at 25 °C and then transferred into PEI/DA codeposition solution for 6 h. Afterward, the modified membranes were washed by DI water for several times and cross-linked by 2.5 wt % GA of aqueous solution for 30 min at 50 °C. Finally, the obtained membranes were rinsed three times and stored in DI water for further characterization and evaluation. For convenience, the aforementioned modified membranes were denoted as M-12C4-x-PEI (x = 0.50 and 0.75). Another group of membranes was successively modified by PEI/DA and 12C4/DA co-deposition solution and further cross-linked, which was denoted as M-PEI-12C4-x (x = 0.50 and 0.75). For comparison, a series of membranes were only modified by PEI/DA or 12C4/DA under the same conditions and they were respectively referred to as M-PEI and M-12C4-x (x = 0.50 and 0.75).

### 2.4. Membrane Characterization

#### 2.4.1. Structure and Morphology Characterization

The chemical structures of membrane surfaces were measured by attenuated total reflectance Fourier transform infrared spectrophotometer (FT-IR, Nicolet 6700, Waltham, MA, USA). Spectra of dried samples were collected in the range of 400–4000 cm^−1^. Semi-quantitative analysis of chemical components was investigated by X-ray photoelectron spectroscopy (XPS) on a spectrometer (Thermo Fischer ESCALAB 250XI, Waltham, MA, USA) with Al Kα excitation radiation (1486.6 eV). The scanning electron microscope (SEM, SU8010 Hitachi, Tokyo, Japan) was used to observe the elements distribution in cross-section of the CEMs. The samples were dried in a freeze dryer for more than 24 h before characterization. 

#### 2.4.2. Ion Exchange Capacity (IEC)

Ion exchange capacity of CEMs was determined by acid–base titration. Pieces of dry CEMs (*W_dry_*, g) were soaked in 25 mL of 1.0 M HCl solution for 48 h to convert all charge sites in membrane matrix into the H^+^ form and then washed with DI water to remove the residual H^+^ on the membrane surface. Subsequently, the membranes were immersed in 25 mL of 0.5 M NaCl solution for 48 h to release H^+^ ions in membrane matrix. The remaining solution was titrated against NaOH solution with a known concentration *(*$c_{NaOH}$, mol·L^−1^) using phenolphthalein as the indicator. The equivalent volume of NaOH was denoted as *V_NaOH_* (mL). The IEC (mmol·g^−1^) value was calculated by Equation (1):

(1) $$IEC=\frac{c_{NaOH}\times V_{NaOH}}{W_{dry}}.$$

#### 2.4.3. Water Uptake

Water uptake (WU) refers to the weight difference after the membrane is fully hydrated, relative to the dry membrane. Before hydrated, membrane samples were dried to constant weight under vacuum and weighed (*W_dry_*, g). Then, samples were immersed in water to reach equilibrium at 25 °C. The excess moisture on membrane surface was wiped with filter paper; then, the wet membrane sample was immediately weighed (*W_wet_*, g). WU was calculated as per Equation (2):

(2) $$WU=\frac{W_{wet}-W_{dry}}{W_{dry}}\times 100\%.$$

### 2.5. Electrochemical Characterization

#### 2.5.1. Surface Area Resistance

**Figure 2:**
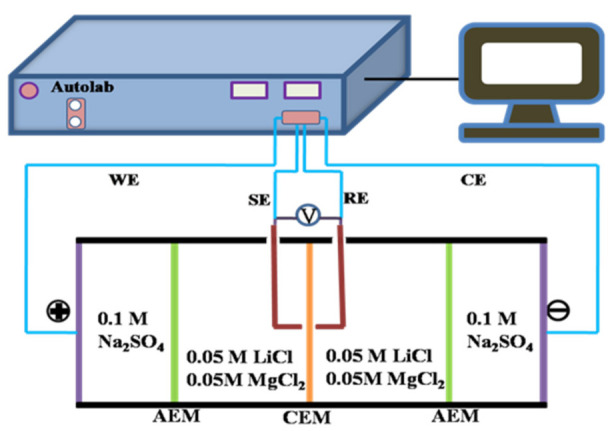
Schematic diagrams of the experimental setup for the measurement of EIS and polarization current–voltage curves.

The surface area resistance (*R*) of the as-prepared CEMs was measured by a custom-designed four-compartment cell and calculated according to electrochemical impedance spectroscopy (EIS) recorded by an Autolab electrochemical workstation (PGSTAT302 N, Metrohm, Herisau, Switzerland). As shown in Figure 2, the measurement was performed according to our published work [28]. In the four-compartment cell, the CEM to be tested was clamped in the middle two compartments filled with 0.5 M chloride salt solution (LiCl and MgCl_2_, respectively), and two pieces of AEMs were placed respectively on both ends of the compartments full of 0.5 M Na_2_SO_4_. The cathode and anode made of titanium coating ruthenium were utilized as the working electrode (WE) and counter electrode (CE), respectively. A couple of saturated Ag/AgCl electrodes, as the reference electrode (RE) and sensitive electrode (SE), were placed on both sides of the tested membrane as closely as possible. EIS was measured with an AC signal of 10 mV amplitude and the frequency ranging from 103 kHz to 100 mHz at room temperature. *R_i_* (Ω·cm^2^) was calculated as follows:

(3) $$R_{i}=\left({\left|Z\right|}_{m}-{\left|Z\right|}_{n}\right)\times S_{m}$$

where ${\left|Z\right|}_{m}$ and ${\left|Z\right|}_{n}$ represent the impedance value with and without the tested CEM, $S_{m}$ is the effective membrane area (7.065 cm^2^) in this setup, and *i* represents electrolyte species.

#### 2.5.2. Current–Voltage test

Current–voltage curves were measured on a four-electrode setup (see the diagram in Figure 2), similar to *R_i_* measurement described above. The DC current was supplied with a scan rate of 250 μA·s^−1^. During the measurement, the electrode solution was 0.1 M Na_2_SO_4_ solution, and the work solution was the mixture of 0.05 M LiCl and 0.05 M MgCl_2_.

#### 2.5.3. Monovalent Cation Selectivity

**Figure 3:**
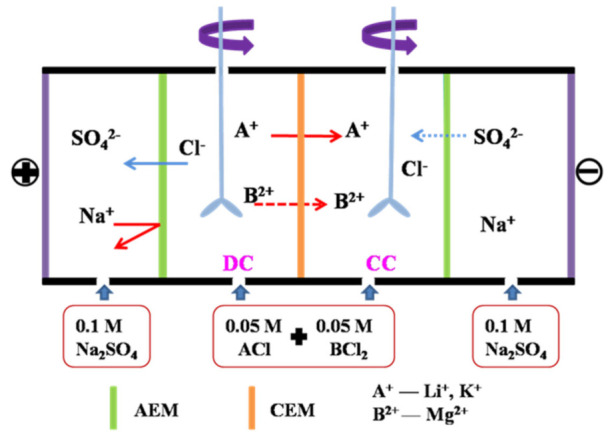
Schematic ED apparatus for the measurement of cation permselectivity for the pristine and modified membranes.

ED experiments were performed in a homemade four-compartment setup to measure the membrane permselectivity (Li^+^/Mg^2+^) under galvanostatic condition at room temperature. The assembly of the device was shown in Figure 3, containing the dilute (DC), the concentrate (CC), and two electrode compartments. The electrode compartments were circulated with 0.1 M Na_2_SO_4_ solution. The DC and CC were both filled with 80 mL of 0.05 M equimolar binary mixtures of chloride salts (Li^+^/Mg^2+^, K^+^/Mg^2+^, K^+^/Li^+^), to avoid the influence of concentration gradients in these compartments. Concentration of the cations in the DC and CC was determined by Cation Chromatography (ICS-1100, Thermo Fisher, Waltham, MA, USA) at 60 min. The flux of the target cation was determined by its concentration change (mol·L^−1^) in the CC as per Equation (4):

(4) $$J_{i}=\frac{V}{A_{m}}\left(\frac{dc_{i}}{dt}\right)$$

where *J_i_* is the flux of cation (*i*) through the CEM (mol·m^−2^·s^−1^), *V* is the volume (L) of electrolyte in the CC (volume change was negligible within a certain time), and *A_m_* is the effective area (19.625 cm^2^) of the tested CEM.

The permselectivity of CEM between *A^m+^* and *B^n+^* (denoted as $P_{B^{n+}}^{A^{m+}}$) was generally defined by the following Equation (5).

(5) $$P_{B^{n+}}^{A^{m+}}=\frac{t_{A^{m+}}/t_{B^{n+}}}{c_{A^{m+}}/c_{B^{n+}}}=\frac{J_{A^{m+}}\cdot c_{B^{n+}}}{J_{B^{n+}}\cdot c_{A^{m+}}}$$

where $t_{A^{m+}}$ and $t_{B^{n+}}$ are the transport numbers of *A^m+^* and *B^n+^* in the membrane phase, $c_{A^{m+}}$ and $c_{B^{n+}}$ are the concentrations (mol·L^−1^) of *A^m+^* and *B^n+^* in the DC during ED, respectively. $J_{A^{m+}}$ and $J_{B^{n+}}$ represent the fluxes (mol·m^−2^·s^−1^) of *A^m+^* and *B^n+^* through the membrane.

Current efficiency (*η*, %) and the special energy consumption (*E_SEC_*, kWh/mol *A^m+^, A^m+^* is the target cation) in the process of ED were calculated according to the Reference [37], as shown in Equations (6) and (7).

(6) $$\eta (\%)=\frac{(n_{t}-n_{0})zF}{N\int_{0}^{t}I(t)dt}\times 100$$

(7) $$E_{SEC}(kWh/molA^{m+})=\frac{I\int_{0}^{t}U(t)dt}{n_{R}}$$

where $n_{t}\;$ and $n_{0}$ are the number of moles of *A^m+^* at time *t* and initial condition in the CC; *z* is the valance of *A^m+^*; *F* is the Faraday constant (96485 C/mol); *I* is the constant current (0.1 A) and *t* is 60 min; *N* is the number of repeating units (*N* = 1); *U* is the detected voltage (V) during the ED process; $n_{R}$ represents the molar number of *A^m^*^+^ increased in the CC.

## 3. Results and Discussions

### 3.1. Chemical Structures and Compositions of the Membrane Surfaces

**Figure 4:**
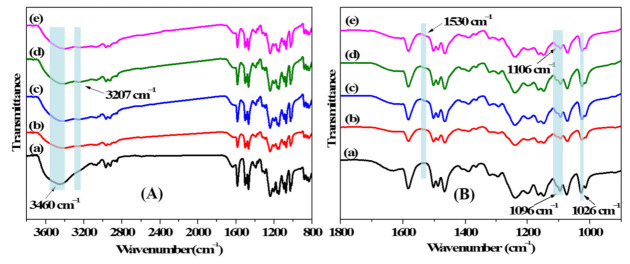
FT-IR spectra of membranes (a) M-0, (b) M-12C4-0.75, (c) M-PEI, (d) M-12C4-0.75-PEI, and (e) M-PEI-12C4-0.75 in (**A**) and (**B**) with different wavenumber ranges.

FT-IR was used to analyze the chemical structures of the membrane surfaces for the pristine and modified membranes, and the spectra are exhibited in Figure 4. The obvious characteristic peaks at 1026 and 1096 cm^−1^ in all of the spectra (a)–(e) are assigned to the symmetric and asymmetric stretching vibrations of the sulfonated group [28,38]. A broad band around 3460 cm^−1^ is ascribed to the stretching vibration of the O-H bond in the sulfonated group and residual moisture in the membrane matrix. The newly developed peaks at 1530 cm^−1^ and 3207 cm^−1^ that emerged in spectra (b)–(e), respectively corresponded to the N–H shearing vibration and stretching vibration derived from PDA, PEI, and 12C4 [39,40]. An additional peak arises at 1106 cm^−1^, which is ascribed to the C–N stretching vibration in PDA, PEI, and 12C4 of the modified layers [41]. In order to further certify the element composition on membrane surfaces, XPS analysis was measured, and the results are displayed in Table 1. In comparison with M–DA, the C/O ratio slightly increases from 3.42 to 3.73, and the O/N ratio significantly increases from 4.30 to 6.01 for M-12C4-0.75, owing to the higher ratio of O element (23.53%) than that of N element (5.88%) in 12C4 molecule. After further codepositing of PEI/DA on the membrane surface for M-12C4-0.75-PEI, a sharply decreased ratio of O/N is mainly attributed to N content of PEI molecule. Compared with M-12C4-0.75-PEI, the increased C/N ratio for M-PEI-12C4-0.75 is derived from the higher C/N ratio in 12C4 than that in PEI.

### 3.2. Physicochemical Properties

**Figure 5:**
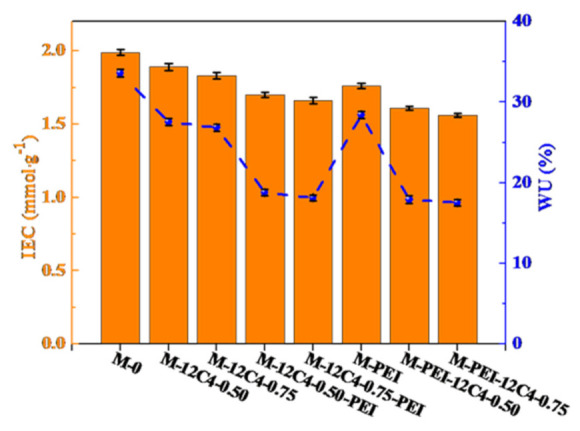
IECs and WUs for the pristine (M-0) and modified membranes (M-PEI, M-12C4-x, M-12C4-x-PEI, M-PEI-12C4-x, x = 0.50 and 0.75).

**Figure 6:**
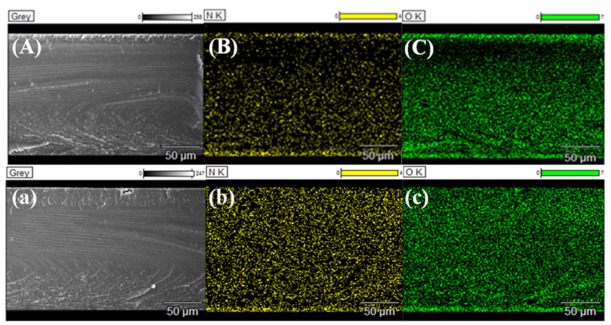
EDX elemental maps in the whole cross-section of modified membranes (**A**) M-PEI-12C4-0.75 and (**a**) M-12C4-0.75-PEI (the bottom end of the section is the modified layer), (**B**,**b**) are the N element mapping and (**C**,**c**) are the O element mapping.

IEC represents the exchangeable ionic sites and sheds light on the ionic conductivity and water absorption in membrane matrix. WU reveals the proportion of the hydrophilic domains in the membrane matrix and influences the ion transportation. IECs and WUs of the pristine and modified membranes are displayed in Figure 5. In general, IEC and WU values of modified membranes almost decrease compared with M-0. The IECs of M-12C4-0.50 and M-12C4-0.75 are respectively 1.89 and 1.83 mmol·g^−1^, while the values gradually fall down to 1.70 and 1.66 mmol·g^−1^ for M-12C4-0.50-PEI and M-12C4-0.75-PEI. From the EDX mapping of the N element in the whole cross-section of M-12C4-0.75-PEI (see Figure 6b), we can see that the distribution of the N element is almost uniform. It can confirm the permeation of codeposition solution (12C4/DA and PEI/DA) to the membrane matrix easily. During the 12C4/DA codeposited reaction, 12C4 and DA gradually permeate into the membrane matrix, and the amine groups mainly from PDA interact with sulfonate groups in the membrane matrix, leading to decreased exchangeable sites [28,42]. The further variation of IECs for M-12C4-x-PEI (x = 0.50 and 0.75) is plausibly attributed to the acid–base interaction between sulfonate groups and DA small molecules in PEI/DA codeposition solution. Furthermore, a secondary modified layer of PEI/DA was coated on the membrane surface, leading to a relatively thicker layer. Indeed, WU of M-12C4-0.75-PEI decreases to 18.15% compared with M-12C4-0.75 (26.86%). It confirms that modified medium occupies abundant free space of the membrane matrix [43]. The IEC of M-PEI is 1.76 mmol·g^−1^; similarly, it drops to 1.56 mmol·g^−1^ for M-PEI-12C4-0.75. Amine groups derived from positive PEI on the membrane surface and DA permeated into the membrane phase contacted with sulfonate groups inherent in the membrane, resulting in lower exchangeable sites. Furthermore, a secondary coating of 12C4/DA not only seriously reduces IECs but also leads to lower WUs for M-PEI-12C4-0.50 (0.75). This should be attributable to the greater accumulation of modified medium in the membrane matrix and thicker modified layers.

### 3.3. Electrochemical Properties

**Figure 7:**
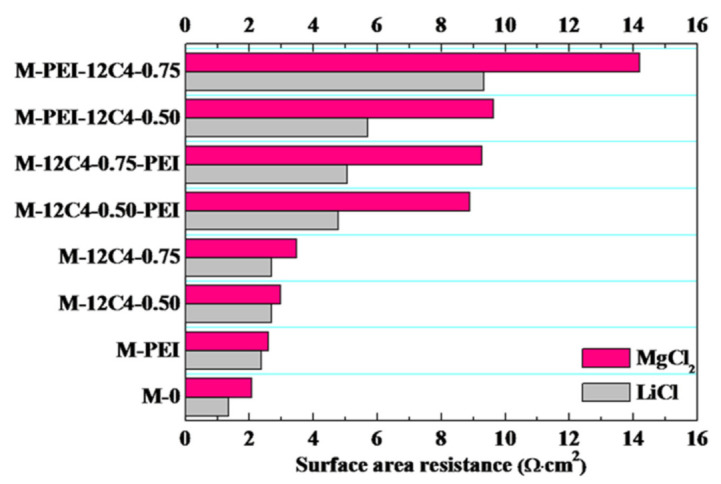
Surface area resistances for the pristine (M-0) and modified membranes (M-PEI, M-12C4-x, M-12C4-x-PEI, M-PEI-12C4-x, x = 0.50 and 0.75).

Surface area resistances (R) of the pristine and modified membranes in LiCl and MgCl_2_ solution were respectively investigated, and the results are presented in Figure 7. It is evident that R values of the modified membranes for one-time deposition (M-PEI, M-12C4-0.50 and M-12C4-0.75) are enhanced relative to M-0 for a given electrolyte solution. Moreover, the R values of two-time coating membranes (M-12C4-x-PEI, M-PEI-12C4-x, x = 0.50 and 0.75) increase remarkably, resulting from the thickened modified layers on the membrane surface and increased modified medium in the membrane matrix. This is in line with the lower IECs and decreased WUs (less free volume), as shown in Figure 5. Furthermore, a great difference of R value occurs in LiCl and MgCl_2_ solution for a specified membrane. In particular, the resistance difference to MgCl_2_ and LiCl is significantly obvious for M-12C4-x-PEI (x = 0.50 and 0.75). The hydrated ion radii of Li^+^ and Mg^2+^ ions are 3.82 and 4.28 Å [44], respectively. In addition, Mg^2+^ shows a lower migration rate and self-diffusion coefficient in aqueous solution [2]. A PEI/DA coating onto the outermost of membrane is more beneficial to Li^+^ transfer than Mg^2+^ than a 12C4/DA coating. As a result, it can reflect the selective transport of Li^+^ vs. Mg^2+^ in a different membrane matrix.

**Figure 8:**
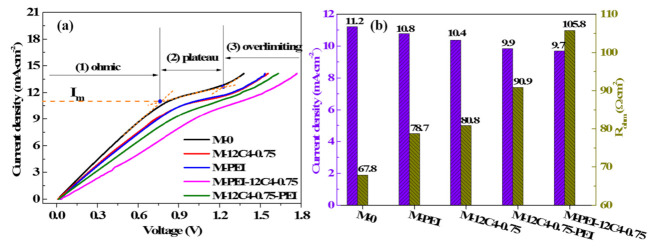
Current density–voltage curves (**a**) and systematic ohmic resistances (**b**) for the pristine (M-0) and modified membranes (M-12C4-0.75, M-PEI, M-PEI-12C4-0.75 and M-12C4-0.75-PEI) in 0.05 M binary mixture of LiCl and MgCl_2_.

Polarization current–voltage (C–V) curves can provide insight into the ion transport behavior of ion exchange membranes over a range of currents [34]. Figure 8a shows the C–V curves of the pristine and several modified membranes measured in the mixture of 0.05 M LiCl and 0.05 M MgCl_2_. All the curves exhibit three typical characteristic regions: ohmic region, plateau region, and over-limiting region [34,45,46]. In the ohmic region, it presents a linear dependence between membrane voltage and current density. Moreover, the ohmic resistance (R_ohm_, Ω·cm^2^) of the membrane system can be served through the inverse of the slope of the ohmic region. Furthermore, a small current increase leads to large improvement of voltage owing to continuous ion depletion in the diffusion boundary layer, which is followed by a plateau occurrence. The limiting current density (*I_m_*, mA·cm^−2^) is determined by the intersection of the ohmic and plateau regions, as the maximum current applied in the membrane system. The membrane voltage further increases linearly with the current density, resulting in an over-limiting region, where water splitting and electroconvection probably appear. 

We can see in Figure 8b that systematic ohmic resistances for all the modified membranes are higher than that of M-0 (67.8 Ω·cm^2^), even reaching the highest value (105.8 Ω·cm^2^) for M-PEI-12C4-0.75. It is in accordance with the results of surface area resistance in Figure 7. On the contrary, *I_m_* of the modified membranes varies from 11.2 to 9.7 mA·cm^−2^, relative to M-0 (see from Figure 8b). These phenomena are consistent with some previous reports [20,28,46,47]. It is primarily attributable that the codeposition products on the membrane surface and permeating into the inner part of membrane matrix suppress cation migration to a certain extent [47]. The heterogeneous membrane surface after coating forms part of non-conductive regions, leading to an earlier and faster salt ion depletion in the conductive regions. Hence, a decreased current density is observable [20].

### 3.4. Evaluation of Monovalent Cation Selectivity

**Figure 9:**
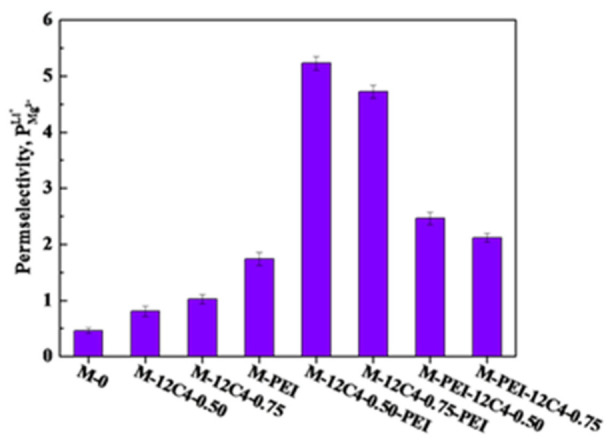
Permselectivity for the pristine (M-0) and modified (M-PEI, M-12C4-x, M-12C4-x-PEI, M-PEI-12C4-x, x = 0.50 and 0.75) membranes in 0.05 M binary mixture of LiCl and MgCl_2_ at current density of 5.0 mA·cm^−2^.

To evaluate the selective separation performance of the modified membranes, ED experiments were measured in 0.05 M binary mixture of LiCl and MgCl_2_. According to the investigation of current–voltage curves, the current density is constant at 5.0 mA·cm^−2^ throughout the tests. Figure 9 shows the permselectivity between Li^+^ and Mg^2+^ of the pristine and modified membranes. M-0 presents a poor permselectivity to Li^+^ ions ($P_{{\mathrm{Mg}}^{2+}}^{{\mathrm{Li}}^{+}}$ = 0.46), which is due to the usually larger attraction of the fixed ion exchange sites toward multivalent cations [48]. With the introduction of a codeposition coating of 12C4/DA, the permselectivity of M-12C4-0.75 to Li^+^ ions is improved up to 1.02. In addition, the modified membrane M-PEI exhibits a higher permselectivity value ($P_{{\mathrm{Mg}}^{2+}}^{{\mathrm{Li}}^{+}}$ = 1.74). Based on these one-time codeposition modified membranes, it is concluded that benzo-12-crown-4 derivatives and PEI respectively introduced on the membrane surface have a remarkably conducive effect on the enhancement of Li^+^ selectivity in the mixture of Li^+^ and Mg^2+^ ions. Furthermore, combining the characteristics of benzo-12-crown-4 and PEI, we have further investigated the selective separation performance to Li^+^ ions of the two-time codeposited membranes. A steep increase in $P_{{\mathrm{Mg}}^{2+}}^{{\mathrm{Li}}^{+}}$ can be apparently observed in comparison to the one-time codeposited membranes, which confirms the positive effects of two-time modification. Moreover, $P_{{\mathrm{Mg}}^{2+}}^{{\mathrm{Li}}^{+}}$ values of M-12C4-0.50-PEI (5.23) and M-12C4-0.75-PEI (4.72) are far higher than those of M-PEI-12C4-0.50 (2.46) and M-PEI-12C4-0.75 (2.12). Obviously, the decoration sequence of benzo-12-crown-4 derivative and PEI has a significant influence on Li^+^ ions separation (the detailed discussion is in Section 3.5).

**Figure 10:**
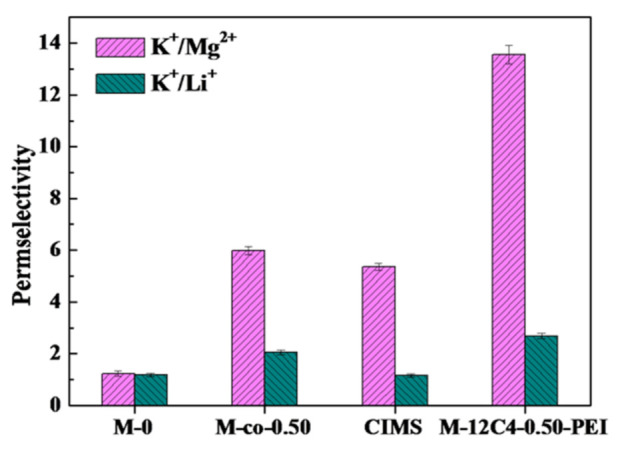
Permselectivity for membranes M-0, M-12C4-0.50-PEI, M-co-0.50 and CIMS in 0.05 M binary mixture of KCl/MgCl_2_ and KCl/LiCl at current density of 5.0 mA·cm^−2^.

To further investigate the separation performance of modified membranes on other cations, M-12C4-0.50-PEI was chosen to be performed by ED in the mixture of K^+^/Mg^2+^ and K^+^/Li^+^. As shown in Figure 10, in K^+^/Mg^2+^ system, M-12C4-0.50-PEI presents remarkable permselectivity to K^+^ ions ($P_{{\mathrm{Mg}}^{2+}}^{K^{+}}$ = 13.56) compared with M-0 ($P_{{\mathrm{Mg}}^{2+}}^{K^{+}}\;$= 1.23), which is far higher than those of M-co-0.50 ($P_{{\mathrm{Mg}}^{2+}}^{K^{+}}\;$= 5.99) and commercial CIMS ($P_{{\mathrm{Mg}}^{2+}}^{K^{+}}\;$= 5.36) reported in our previous work in the same test condition [28]. It can be mainly due to the strong electrostatic repulsion effect of PEI coated on the outermost of the membrane. Whereas, for the separation of K^+^/Li^+^, $P_{K^{+}}^{{\mathrm{Li}}^{+}}$ reaches 2.69 for M-12C4-0.50-PEI, which is much higher than those of M-0 (1.18), M-co-0.50 (2.05) and CIMS (1.16). It can be speculated that the dense modified membrane structure restrains the transportation of cations with larger hydrated radii through a pore-size sieving effect.

### 3.5. Analysis of the Effects of Modification Sequence on Cation Permselectivity

From the investigation of cation permselectivity for the CEMs stated above, we can see the two-time codeposition modified membranes M-12C4-0.50-PEI and M-12C4-0.75-PEI achieve distinct selective separation between binary mixture cations. It is plausibly attributed to three synergistic effects: electrostatic repulsion, ion–dipole interaction between Li^+^ ions and benzo-12-crown-4 derivatives, as well as pore-size sieving effect. As is expected, the introduction of benzo-12-crown-4 is to provide facile ion channels for Li^+^ ions, while the presence of positively charged PEI is to inhibit Mg^2+^ ions migration through the membrane via strong electrostatic repulsion toward multivalent cations.

**Figure 11:**
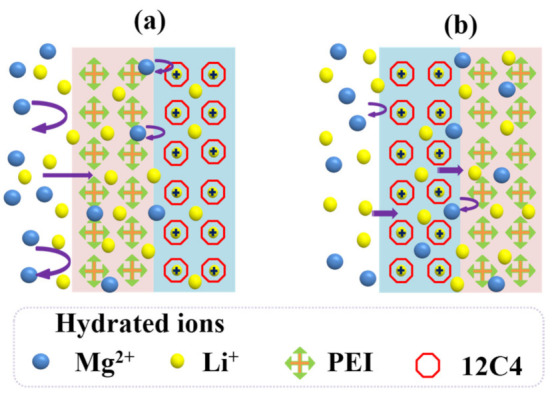
Analysis for the selective permeation of cations (Li^+^ and Mg^2+^) through modified layers of membranes (**a**) M-12C4-0.50-PEI and (**b**) M-PEI-12C4-0.50.

From the results of Li^+^/Mg^2+^ separation for one-time codeposited membranes (see Figure 9), it is concluded that the electrostatic repulsion of the PEI coating is more effective than the ion–dipole interaction of 12C4 decorating. Similarly, for the two-time codeposited membranes, PEI coated on the outermost layer plays a dominant role to achieve outperforming permselectivity to Li^+^ ions; $P_{{\mathrm{Mg}}^{2+}}^{{\mathrm{Li}}^{+}}$ values are respectively 2.46 and 5.23 for M-PEI-12C4-0.50 and M-12C4-0.50-PEI. Figure 11 presents the different processes of cation selective permeation (Li^+^ and Mg^2+^) through modified layers of membranes M-12C4-0.50-PEI and M-PEI-12C4-0.50. Cations in the bulk of the dilute side migrate directionally to the interface of modified CEM under the electric field force. For M-12C4-0.50-PEI (see Figure 11a), abundant positively charged amine groups of PEI on the secondary modified layer (PEI/DA) show stronger electrostatic repulsion toward major Mg^2+^ ions relative to Li^+^ ions. When the remaining cations migrate into the first modified layer (12C4/DA), part of Li^+^ ions are first dehydrated and then complexed with ether ring of 12C4. The complexing formations of 12C4 ~Li^+^ present positive, and they can further suppress the transportation of partial Mg^2+^ ions through electrostatic repulsion. Under the driving of galvanostatic electric field, Li^+^ ions in complexed form can gradually pass through the membrane [49]. The last but not the least, as stated aboved, modified medium occupies abundant free space of the membrane matrix, which results in lower WUs (see Figure 5), higher R_ohm_ (see Figure 7), and denser structures. This pore-size sieving effect is conducive to the passing of cations with smaller hydrated radii. However, among the cations on the membrane surface for M-PEI-12C4-0.50, dehydrated Li^+^ ions are prone to interact with 12C4 on the outer modified layer (12C4/DA), which would slow down the motion of Li^+^ ions; meanwhile, the migration of Mg^2+^ ions is also partially affected by the positive complexing. Moreover, in addition to reacting with DA on the first modified layer (PEI/DA), the remaining amine groups of PEI are inclined to react with DA from the secondary deposition solution (12C4/DA). It can be speculated that the free positively charged PEI shows relatively weak electrostatic repulsion toward cations. Combined with the electrodialytic performance of one-time modified membranes (M-PEI and M-12C4-0.50), it is not difficult to conclude the prominent electrodialytic permselectivity to Li^+^ ions in the Li^+^/Mg^2+^ system for M-12C4-0.50-PEI.

**Figure 12:**
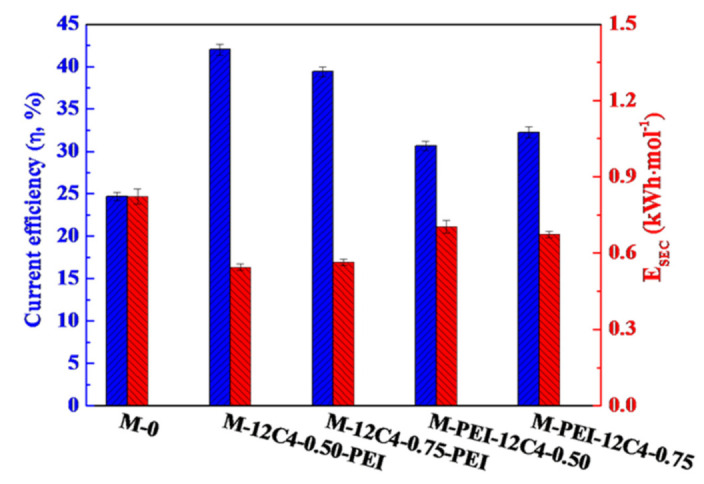
Current efficiency (η) and energy consumption (E_SEC_) of the pristine (M-0) and modified (M-12C4-x-PEI, M-PEI-12C4-x, x = 0.50 and 0.75) membranes in 0.05 M binary mixture of LiCl and MgCl_2_ at a current density of 5.0 mA·cm^−2^.

In addition, energy consumption (E) and current efficiency (η) are significant parameters to evaluate the performance of membranes. As shown in Figure 12, M-PEI-12C4-0.50 displays increased E_SEC_ and decreased η values in comparison with M-12C4-0.50-PEI, which is attributed to the reduction in membrane conductivity (lower IECs and WUs shown in Figure 5). A thin rigid coating on membrane surface affects the microstructure of membrane; thus, partial ionic transport is retarded [25,50]. R_LiCl_ and R_MgCl2_ of M-PEI-12C4-0.50 are respectively higher than those of M-12C4-0.50-PEI (see in Figure 7), respectively, which manifests that a more compact membrane structure for M-PEI-12C4-0.50 suppresses the migration of Li^+^ and Mg^2+^ ions. 

Furthermore, due to the smaller hydrated radii of K^+^ ions compared with Li^+^ ions, for the separation of K^+^/Mg^2+^, the synergistic effects of electrostatic repulsion and pore-size sieving effect are more prominent for M-12C4-0.50-PEI.

## 4. Conclusions

A series of novel monovalent cation perm-selective membranes have been fabricated based on sulfonated polysulfone (SPSF) membrane. By changing the codeposition sequence of PEI/DA and 12C4/DA on the membrane surface, significant differences present in the physicochemical and electrochemical properties as well as the electrodialytic performance of the modified membranes. M-12C4-0.50-PEI exhibited significantly prominent permselectivity to Li^+^ ions in the Li^+^/Mg^2+^ system, which possibly arises from the synergistic effects of electrostatic repulsion (positively charged PEI), pore-size sieving (distribution of modified ingredients) and specific interaction effect (12C4 ~Li^+^). A higher permselectivity to K^+^ ions of M-12C4-0.50-PEI is mainly attributable to the strong electrostatic repulsion and pore-size sieving effect, in comparison with M–0. This work can provide a potential approach in separation of specific cations.

## Figures and Tables

**Table 1:** Chemical composition of the pristine (M-0) and modified (M-DA, M-12C4-0.75, M-12C4-0.75-PEI, and M-PEI-12C4-0.75) membranes surface from XPS spectra.

Materials/Membranes	C (%)	O (%)	N (%)	S (%)	C/O	C/N	O/N
DA (theoretical)	72.73	18.18	9.09		4.00	8.00	2.00
PEI (theoretical)	66.67		33.33			2.00	
12C4 (theoretical)	70.59	23.53	5.88		3.00	12.00	4.00
M-0	76.39	20.08		3.53	3.80		
M-DA	71.95	21.06	4.90	2.09	3.42	14.68	4.30
M-12C4-0.75	74.88	20.05	3.34	1.73	3.73	22.45	6.01
M-12C4-0.75-PEI	76.62	18.90	3.67	0.82	4.05	20.90	5.16
M-PEI-12C4-0.75	78.77	17.46	3.08	0.69	4.51	25.56	5.66

## Data Availability

Not applicable.
